# Supplementary material for: Histology-informed spatial domain identification through multi-view graph convolutional networks
Source: PLoS Comput Biol. 2026 Jun 1;22(6):e1014281. doi: 10.1371/journal.pcbi.1014281 (PMC13225418; doi:10.1371/journal.pcbi.1014281)
Supplement: S1 Fig — (DOCX) [file pcbi.1014281.s001.docx]

***S1 Fig: Identification of spatial domains for DLPFC by STESH with no histological images.*** *The analysis was performed on the following tissue slides: 151507, 151508, 151509, 151510, 151669, 151670, 151671, 151672, 151673, 151674, 151675, and 151676.*
