# Supplementary material for: Histology-informed spatial domain identification through multi-view graph convolutional networks
Source: PLoS Comput Biol. 2026 Jun 1;22(6):e1014281. doi: 10.1371/journal.pcbi.1014281 (PMC13225418; doi:10.1371/journal.pcbi.1014281)
Supplement: S8 Fig — (DOCX) [file pcbi.1014281.s008.docx]

***S8 Fig: Annotation of DLPFC layers and identification of spatial domains by multiple methods in slide 151671.*** *The DLPFC layers of tissue slide 151671 were annotated by Maynard et al. Spatial domains were identified using the following methods: STESH, GraphST, Spatial-MGCN, SEDR, DeepST, STAGATE, SpaGCN, BayesSpace, stLearn, spaceFLOW, and Leiden.*
