# Supplementary material for: Histology-informed spatial domain identification through multi-view graph convolutional networks
Source: PLoS Comput Biol. 2026 Jun 1;22(6):e1014281. doi: 10.1371/journal.pcbi.1014281 (PMC13225418; doi:10.1371/journal.pcbi.1014281)
Supplement: S13 Fig — (DOCX) [file pcbi.1014281.s013.docx]

***S13 Fig: Analysis of gene numbers in brain cortex pathways for STESH, Spatial-MGCN, and GraphST in slice 151672.*** *(a) Absolute differences in gene numbers (color intensity corresponds to the magnitude of the difference). (b) Predicted gene numbers versus ground truth values. (c) Gene numbers of axon and postsynapse pathways across different brain cortex regions.*
